# Wnt1 induces osteoblastic changes in a well‐established osteolytic skeletal metastatic model derived from breast cancer

**DOI:** 10.1002/cnr2.1909

**Published:** 2023-10-15

**Authors:** Aya Sugyo, Atsushi B. Tsuji, Hitomi Sudo, Yoshiya Sugiura, Mitsuru Koizumi, Tatsuya Higashi

**Affiliations:** ^1^ Experimental Nuclear Medicine Group, Department of Molecular Imaging and Theranostics Institute for Quantum Medical Science, National Institutes for Quantum Science and Technology Chiba Japan; ^2^ Department of Pathology Toho University Sakura Medical Center Sakura Japan; ^3^ Department of Nuclear Medicine Cancer Institute Hospital of Japanese Foundation for Cancer Research Tokyo Japan

**Keywords:** osteoblastic bone metastasis, osteolytic bone metastasis, SATB2, Wnt signaling pathway, Wnt1

## Abstract

**Background:**

Osteoblastic skeletal metastasis is frequently observed in prostate cancer. An effective therapy has not been developed due to the unclear molecular mechanism. The Wnt family is involved in various biological phenomena including bone metabolism. There is no direct evidence that the family causes osteoblastic skeletal metastasis.

**Aims:**

The present study aims to evaluate whether overexpressed Wnt induces osteoblastic bone metastasis in a well‐established osteolytic bone metastatic model.

**Methods and Results:**

The breast cancer‐derived 5a‐D‐Luc‐ZsGreen cells were transfected with Wnt1, Wnt3A, and Wnt5A expression vectors, producing stably highly expressing cells. These cells were intracardially transplanted in nude mice. Bone metastasis development was confirmed by fluorescence imaging. Hind‐limb bones including metastasis were dissected and visualized through micro‐CT imaging. After imaging, sections were stained with hematoxylin and eosin (H&E), and immunohistochemically stained with an anti‐SATB2 antibody. Luminescent imaging confirmed mice with bone metastases in the hind limbs. Micro‐CT imaging found an osteoblastic change only in bone metastasis of mice transplanted with Wnt1‐expressing cells. This was confirmed on H&E‐stained sections. SATB2 immunostaining showed differentiated osteoblasts were at the site of bone metastases in the diaphysis. SATB2 in the Wnt/β‐catenin pathway activated by overexpressed Wnt1 could induce osteoblastic change.

**Conclusion:**

Our findings provided direct evidence Wnt1 is involved in osteoblastic bone metastasis development. Our model would be a powerful tool for further elucidating molecular mechanisms underlying the disease and developing effective therapies.

## 1 INTRODUCTION

Up to 90% of prostate cancers and about 65%–75% of breast cancers have bone metastases,^1^ causing complications such as severe pain, bone fractures, spinal cord compression, and myelosuppression.^1^, ^2^ They significantly impair patients' quality of life and worsen their prognosis.^1^, ^2^, ^3^ Bone metastases are broadly classified into the osteoblastic type, which is common in prostate cancer,^2^, ^3^ and the osteolytic type, which is common in breast cancer.^1^, ^3^

The osteolytic bone metastasis animal models have been well‐established,^4^, ^5^ enhancing our understanding of the molecular mechanism and allowing new therapeutic methods to be developed.^1^, ^6^, ^7^, ^8^ However, for osteoblastic bone metastasis, the molecular mechanism remains unclear, inhibiting the development of specific therapeutic options. Bone metastasis mutually transitions between osteogenesis and osteolysis, and the state of balance between bone resorption and bone formation appears as a type.^9^, ^10^ To elucidate the molecular mechanism, an animal model of osteoblastic bone metastasis is required.

The Wnt family is a candidate factor associated with developing osteoblastic bone metastasis because Wnt1, Wnt3A, and Wnt5A play a major role in the regulation of skeletal development and/or bone formation.^11^ Its signal pathway is activated in many cancers.^8^, ^12^, ^13^, ^14^, ^15^, ^16^, ^17^ Unfortunately, there is no direct evidence that they are related to osteoblastic bone metastasis to date.

5a‐D‐Luc‐ZsGreen is an osteolytic bone metastasis model, derived from the breast cancer cell line MDA‐MB‐231.^5^, ^18^, ^19^ In these cells, the expression levels of Wnt1, Wnt3A, and Wnt5A are low, and there are no mutations in related molecules.^20^, ^21^, ^22^, ^23^ The 5a‐D‐Luc‐ZsGreen induces only osteolytic changes at metastatic sites but not osteoblastic changes.^5^, ^18^, ^19^ These characteristics are suitable to determine whether an osteoblastic lesion is induced by Wnt.

Here, we evaluated whether overexpressed Wnt1, Wnt3A, and Wnt5A induce osteogenic bone metastasis in the 5a‐D‐Luc‐ZsGreen mouse model. Three different expression vectors for Wnt1, Wnt3A, and Wnt5A were transfected into 5a‐D‐Luc‐ZsGreen cells, and stable expression cell clones were established. These clones were injected into the left cardiac ventricle of mice, and bone metastasis development was monitored by temporal bioluminescence imaging. The bone metastasis was characterized by micro‐CT imaging and histologic analysis.

## 2 MATERIALS AND METHODS

### 2.1 Cell culture and transfection

5a‐D‐Luc‐ZsGreen cells^5^ were maintained in a DMEM medium (044–29 765, FUJIFILM Wako Chemicals, Osaka, Japan) supplemented with 5% FBS (173 012, Sigma‐Aldrich, St. Louis, MO) in a humidified incubator maintained at 37 °C with 5% CO_2_. The pcDNA‐Wnt1 (35 905, Addgene, Watertown, MA), pcDNA‐Wnt3A (35 908, Addgene), and pcDNA‐Wnt5A (35 911, Addgene) vectors with the neomycin‐resistant gene were purchased. They were subcloned into pcDNA3.1/Hygro plasmids (V87020, Thermo Fisher Scientific, Waltham, MA, USA). These vectors were transfected into the 5a‐D‐Luc‐ZsGreen cells using Lipofectamine 2000 Transfection Reagent (11 668 027, Thermo Fisher Scientific). The cells were cultured in DMEM with 5% FBS and 0.4 mg/mL hygromycin B (09287‐84, Nacalai Tesque, Kyoto, Japan), and eight subclones were subsequently isolated in each transfectant stably expressing Wnt1, Wnt3A, and Wnt5A protein (5a‐D‐Luc‐ZsGreen‐Wnt1, 5a‐D‐Luc‐ZsGreen‐Wnt3A, and 5a‐D‐Luc‐ZsGreen‐Wnt5A).

### 2.2 Real‐time quantitative RT‐PCR

First‐strand cDNA was synthesized from the subclones of stably expressing Wnt1, Wnt3A, and Wnt5A using a FastLane Cell cDNA kit (215 011, Qiagen, Hilden, Germany). Real‐time RT‐PCR was performed in triplicate using predesigned and preoptimized TaqMan probes to detect Wnt1, Wnt3A, Wnt5A, and 18S rRNA (Applied Biosystems, Foster City, CA). Gene expression levels in each sample were normalized against 18S rRNA expression.

### 2.3 Animal models

For intracardiac injection, female nude mice (6‐week‐old; CLEA Japan, Tokyo, Japan) were anesthetized by inhalation of 3% isoflurane and injected with 5a‐D‐Luc‐ZsGreen, 5a‐D‐Luc‐ZsGreen‐Wnt1 (Wnt1‐2), 5a‐D‐Luc‐ZsGreen‐Wnt3A (Wnt3A‐2), and 5a‐D‐Luc‐ZsGreen‐Wnt5A (Wnt5A‐3) cells (1 × 10^5^ cells) in 100 μL D‐PBS(−) (200 mg/L KCL, 8000 mg/L NaCl, 1150 mg/L Na_2_HPO_4_, 200 mg/L KH_2_PO_4_; 045‐29 795, FUJIFILM Wako Chemicals) into the left cardiac ventricle as described previously.^4^, ^19^

### 2.4 In vivo bioluminescence imaging

Mice were injected with 2.5 mg D‐luciferin potassium salt (126‐05116, FUJIFILM Wako Chemicals) into a tail vein, and the bioluminescence images were acquired with the IVIS Lumina (Xenogen, Alameda, CA) under isoflurane anesthesia (3%, 119701G1106, VIATRIS, Canonsburg, PA). Mean radiance (photon/s/cm^2^/sr) in the region of interest (ROI) drawn over bioluminescence signals was measured using Living Image software (Xenogen).

### 2.5 Micro‐CT imaging

In the first study, we set the endpoint at 42 days after cardiac injection or when the mice lost 20% of their body weight. In the second one, 28 days after cardiac injection was set as the endpoint to reduce pain due to the progression of osteolytic bone metastasis. After euthanasia by excess isoflurane, dissected hind‐limb bones were fixed in 10% formaldehyde natural buffer solution (37152‐51, Nacalai Tesque) and were scanned by micro‐CT imaging using the CosmoScan GX II system (Rigaku, Tokyo, Japan) with the following parameters: 90 kV of x‐ray tube voltage, and 88 μA of x‐ray tube current.

### 2.6 Histologic analysis

After CT imaging, the fixed bones were embedded in paraffin and cut into 1‐μm thick sections. Three normal lesions, three osteolytic lesions, and five Wnt1 lesions were sliced. Approximately 120 serial sections were sliced. The tumor sections were deparaffinized every 20 slides and stained with hematoxylin and eosin (H&E; 6187‐4P for hematoxylin, 9135‐4P for eosin, Sakura Finetek, Tokyo, Japan) to confirm bone metastasis sites. Adjacent sections of slides in which sites of bone metastasis were confirmed were selected, and immunohistochemically staining was performed according to the following protocol. Deparaffinized tissue sections were pre‐treated in 0.01 mol/L sodium citrate buffer (pH 6; 31 404‐15, Nacalai Tesque) for 20 min. The sections were exposed to 3% hydrogen peroxide (084‐07441, FUJIFILM Wako Chemicals) to eliminate endogenous peroxidase activity and treated with Protein Block Serum‐Free blocking reagent (X0909; Agilent, Santa Clare, CA). The sections were then incubated with anti‐SATB2 antibody (1:400; ab51502; Abcam, Cambridge, UK) overnight at 4°C. After washing, sections were incubated for 30 min with goat polyclonal antibodies to mouse IgG H&L (ab2147879; Abcam) as the secondary antibody. After washing, the antibodies were visualized using diaminobenzidine (25985‐50, Nacalai Tesque). The number of SATB2‐positive cells was counted in six fields (420 μm × 230 μm) of the epiphysis and diaphysis without metastases. In the diaphysis with metastasis, positive cells were counted in eight fields of osteolytic type 5a‐D‐Luc‐ZsGreen and thirteen fields of osteoblastic type 5a‐D‐Luc‐ZsGreen‐Wnt1.

### 2.7 Statistical analysis

Data are expressed as the means ± standard deviation. Statistical analysis was performed using GraphPad Prism 9. SATB2‐staining data were analyzed by one‐way ANOVA with Turkey's multiple comparison test.

## 3 RESULTS

### 3.1 Wnt strong‐expressing cell lines

**FIGURE 1:**
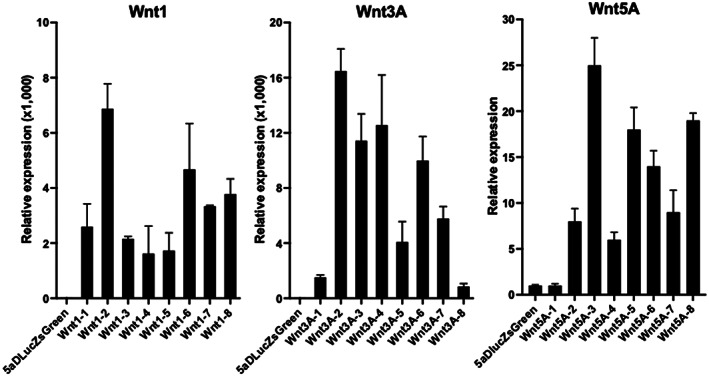
Gene expression analysis of stably Wnt‐expressing subclones. They were produced by transfection of Wnt1, Wnt3A, and Wnt5A vectors into 5a‐D‐Luc‐ZsGreen cells. Wnt expression determined by quantitative real‐time RT‐PCR is normalized to that of 5a‐D‐Luc‐ZsGreen cells.

Wnt expression analysis of stable Wnt‐expressing subclones found the strongest expression subclones, Wnt1‐2 (6.9 × 10^3^‐fold), Wnt3A‐2 (1.6 × 10^4^‐fold), and Wnt5A‐3 (25‐fold), compared with the parental 5a‐D‐Luc‐ZsGreen cells by real‐time RT‐PCR (Figure 1). They were selected for the following experiment, and each subclone was named 5a‐D‐Luc‐ZsGreen‐Wnt1, 5a‐D‐Luc‐ZsGreen‐Wnt3A, and 5a‐D‐Luc‐ZsGreen‐Wnt5A, respectively.

### 3.2 The first experiment to evaluate bone metastasis of three Wnt‐expressing cells

**FIGURE 2:**
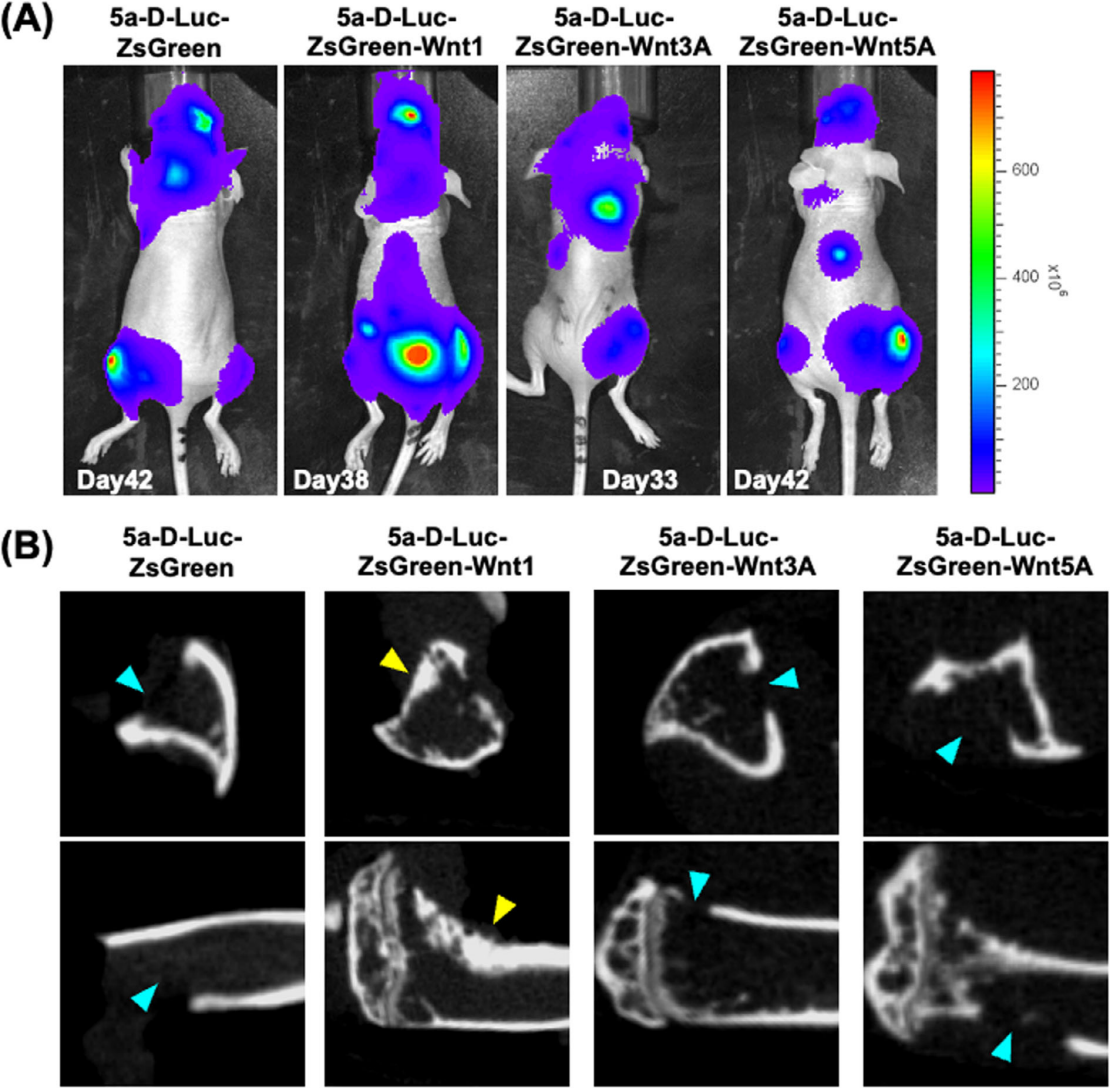
Bioluminescence and micro‐CT imaging in the first experiment. (A) Bioluminescence images of metastasis of 5a‐D‐Luc‐ZsGreen‐Wnt1, 5a‐D‐Luc‐ZsGreen‐Wnt1, 5a‐D‐Luc‐ZsGreen‐Wnt3A, and 5a‐D‐Luc‐ZsGreen‐Wnt5A. The day of imaging is at the bottom of each image. (B) Micro‐CT images of bone metastasis in tibiae of mice injected with 5a‐D‐Luc‐ZsGreen, 5a‐D‐Luc‐ZsGreen‐Wnt1, 5a‐D‐Luc‐ZsGreen‐Wnt3A, and 5a‐D‐Luc‐ZsGreen‐Wnt5A. Blue arrowheads indicate osteolytic lesions. Yellow arrowheads indicate osteoblastic lesions.

5D‐Luc‐ZsGeen (10 mice), 5D‐Luc‐ZsGeen‐Wnt1 (15 mice), 5D‐Luc‐ZsGeen‐Wnt3A (15 mice), and 5D‐Luc‐ZsGeen‐Wnt5A (15 mice) cells were injected into the left cardiac ventricle. The endpoint was set at a maximum of 42 days or when the mice had lost ~20% of their body weight. Bone metastases to the hindlimb were confirmed by luminescence imaging (Figure 2A). Bone metastasis was observed in mice of intracardiac injection with 5a‐D‐Luc‐ZsGreen (5 of 10 mice), 5a‐D‐Luc‐ZsGreen‐Wnt1 (7 of 15 mice), 5a‐D‐Luc‐ZsGreen‐Wnt3A (8 of 15 mice), and 5a‐D‐Luc‐ZsGreen‐Wnt5A (7 of 15 mice) cells (Table 1). Hind limbs observed bone metastases were dissected and conducted by CT imaging. In 5a‐D‐Luc‐ZsGreen‐Wnt1, CT imaging revealed not only osteolytic features but also osteoblastic bone metastases (2 of 7 animals, 28.6%) (Figure 2B). No osteoblastic image was observed in bones with the other three 5a‐D‐Luc‐ZsGreen, 5a‐D‐Luc‐ZsGreen‐Wnt3A, and 5a‐D‐Luc‐ZsGreen‐Wnt5A metastases (Figure 2B; Table 1).

**TABLE 1:** Experiment 1: Intracardiac inoculation of parent cells and three Wnt‐expressing cell lines.

	Bone metastasis bioluminescence	Osteolytic change μCT	Osteoblastic change μCT
5a‐D‐Luc‐ZsGreen	5	5 (100%)	0 (0%)
5a‐D‐Luc‐ZsGreen‐Wnt1	7	7 (100%)	2 (28.6%)
5a‐D‐Luc‐ZsGreen‐Wnt3A	8	8 (100%)	0 (0%)
5a‐D‐Luc‐ZsGreen‐Wnt5A	8	8 (100%)	0 (0%)

*Note*: The endpoint was a 20% reduction in body weight of the mice or a maximum of 42 days.

**FIGURE 3:**
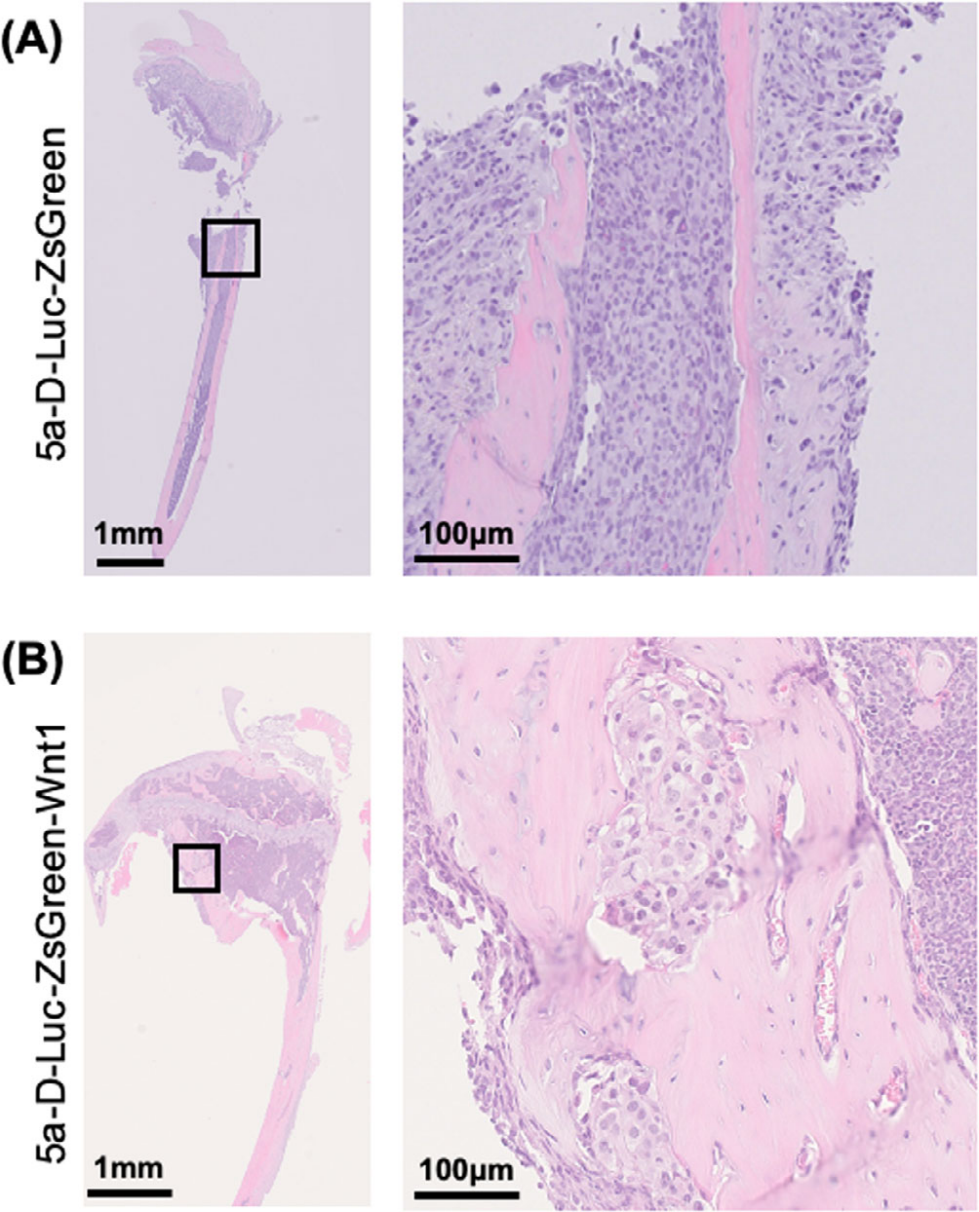
Hematoxylin and eosin staining of bone metastasis lesions of mice in the 1st experiment. (A) A tibia of a mouse with osteolytic bone metastasis after intracardiac injection of 5a‐D‐Luc‐ZsGreen cells. (B) A tibia of a mouse with osteoblastic bone metastases after intracardiac injection of 5a‐D‐Luc‐ZsGreen‐Wnt1 cells.

Sections of 5a‐D‐Luc‐ZsGreen‐ and 5a‐D‐Luc‐ZsGreen‐Wnt1‐metastasized tibia were H&E‐stained (Figure 3). In the 5a‐D‐Luc‐ZsGreen bone metastasis model, the bone marrow cavity was almost completely replaced by metastatic tumor cells and formed osteolysis bone tumor mass (Figure 3A). In the 5a‐D‐Luc‐ZsGreen‐Wnt1 bone metastasis model, tumor cells invaded the diaphysis and formed osteoblastic tumors (Figure 3B).

### 3.3 The second experiment to evaluate bone metastasis of Wnt1‐expressing cells

**FIGURE 4:**
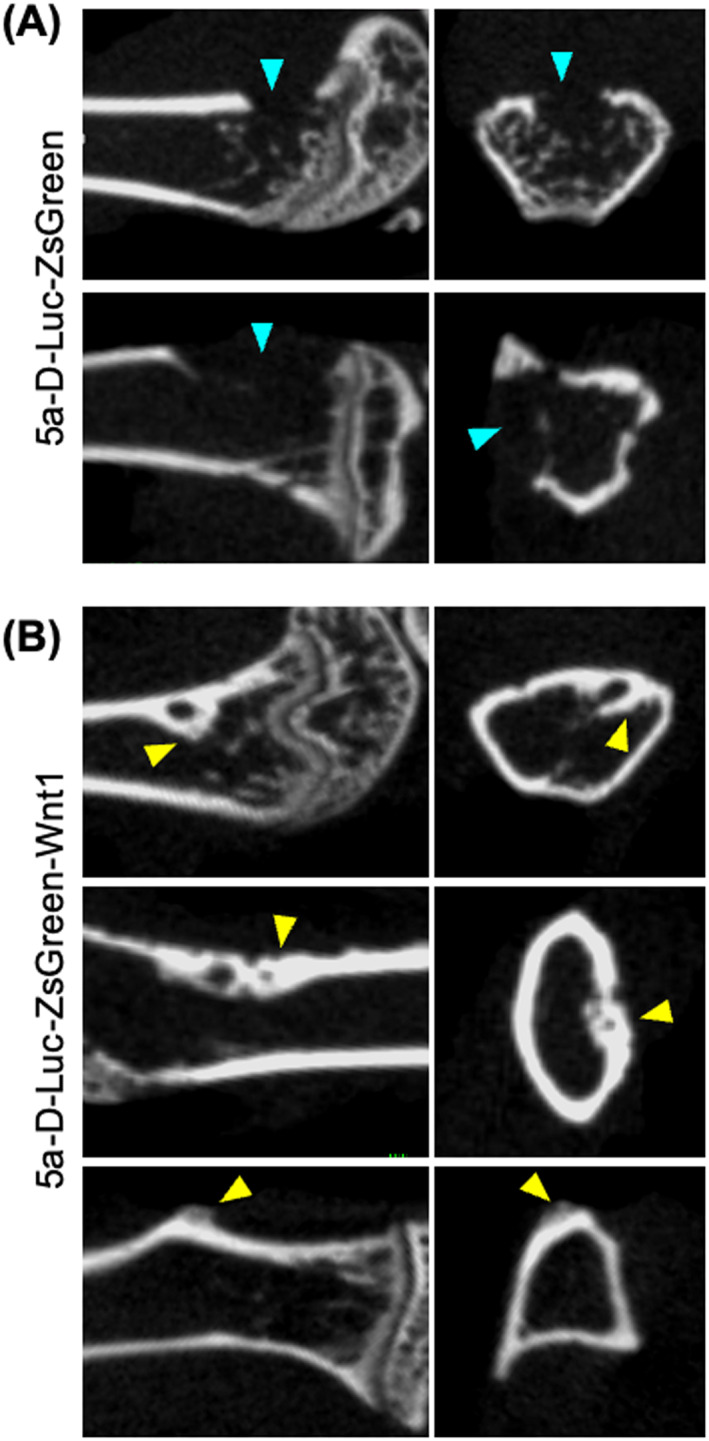
Micro‐CT images of bone metastasis at day 28 after intracardiac inoculation at the second experiment. (A) Osteolytic bone metastasis of mice inoculated with 5a‐D‐Luc‐ZsGreen cells. (B) Osteoblastic bone metastasis of mice inoculated with 5a‐D‐Luc‐ZsGreen‐Wnt1 cells. Blue arrowheads indicate osteolytic lesions. Yellow arrowheads indicate osteoblastic lesions.

To confirm the first experiment, another experiment was conducted in 5D‐Luc‐ZsGeen and 5D‐Luc‐ZsGeen‐Wnt1. The endpoint was set at 28 days to reduce pain due to the progression of osteolytic bone metastasis because the osteoblastic bone metastasis was observed around 28 days in the first experiment. 5a‐D‐Luc‐ZsGreen‐Wnt1 cells with osteoblastic bone metastases or 5a‐D‐Luc‐ZsGreen cells with osteolytic bone metastases were intracardially injected. After bone metastases were confirmed in the hind limbs by luminescence imaging, they were sampled. In 3 of 11 in 5a‐D‐Luc‐ZsGreen and 4 of 27 in 5a‐D‐Luc‐ZsGreen‐Wnt1, no metastatic bone change was observed by micro‐CT imaging (Table 2). In 8 of 11 (72.7%) mice injected with 5a‐D‐Luc‐ZsGreen cells, CT imaging revealed osteolytic bone metastases in the femur (Figure 4A, upper panel) or tibia (Figure 4A, lower panel), but no osteoblastic bone metastasis was observed (Table 2). In mice injected with 5a‐D‐Luc‐ZsGreen‐Wnt1 cells, not only osteolytic bone metastases were observed in 23 of 27 mice (85.2%) but also containing osteoblastic components were found in 13 of 27 mice (48.1%) (Table 2). The osteoblastic bone metastases were observed on the internal side of the femur (Figure 4B, upper panel), the diaphysis of the femur (Figure 4B, middle panel), and the lateral side of the tibia (Figure 4B, lower panel). All the CT images of osteoblastic bone metastases are provided in Figure S2.

**TABLE 2:** Experiment 2: Intracardiac inoculation with the parent cells and the Wnt1‐expressing cell line.

	Bone metastasis bioluminescence	No change μCT	Osteolytic change μCT	Osteoblastic change μCT
5a‐D‐Luc‐ZsGreen	11	3*	8 (72.7%)	0 (0%)
5a‐D‐Luc‐ZsGreen‐Wnt1	27	4*	23 (85.2%)	13 (48.1%)

*Note*: The endpoint was 28 days after intracardiac injection with 5a‐D‐Luc‐ZsGreen or 5a‐D‐Luc‐ZsGreen‐Wnt1 cells.

* No osteolytic or osteogenic lesions, only intratrabecular invasion.

### 3.4 SATB2 immunohistochemical analysis

**FIGURE 5:**
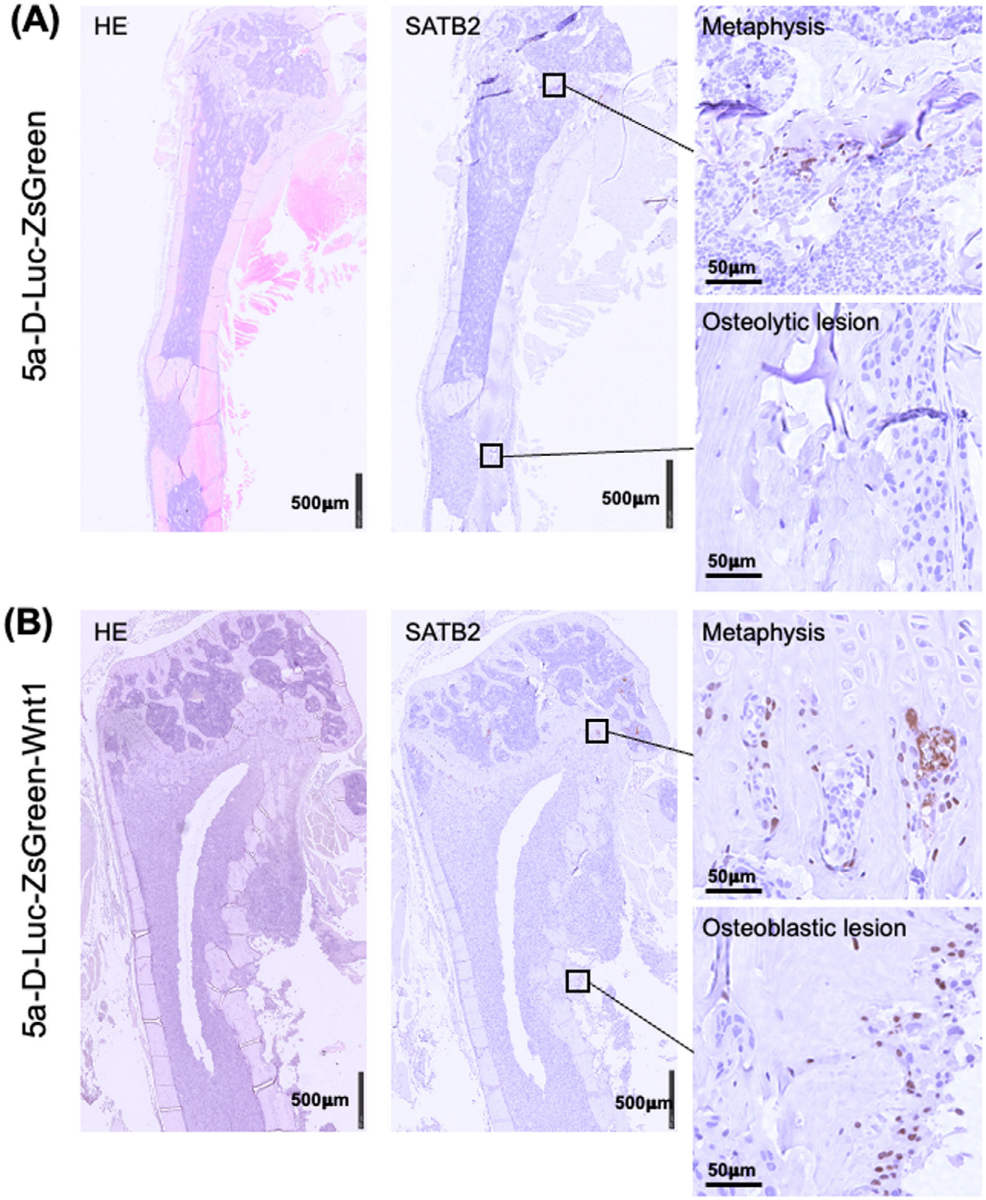
Hematoxylin and eosin and SATB2‐immunohistological staining of bones in mice at the 2nd experiment. (A) Osteolytic bone metastasis of a mouse inoculated with 5a‐D‐Luc‐ZsGreen cells. (B) Osteoblastic bone metastasis of a mouse inoculated with 5a‐D‐Luc‐ZsGreen‐Wnt1 cells.

SATB2, an osteoblast differentiation marker,^24^, ^25^ was stained to determine mature osteoblasts. The representative images are shown in Figure 5 for 5a‐D‐Luc‐ZsGreen and 5a‐D‐Luc‐ZsGreen‐Wnt1 mice and Figure S2 for normal mice. Table 3 shows the number of SATB2‐positive cells in bones. In all mice, there seem many SATB2‐positive cells in the metaphysis and a few in the diaphysis (Figure 5A,B and Figure S2). The quantitative analysis showed the number of SATB2‐positive cells was around 50 cells in the metaphysis and around 10 cells in the diaphysis (Table 3). There is no significant difference in the positive cells although the number in 5a‐D‐Luc‐ZsGreen‐Wnt1 tends to be lower than the others (Table 3). In the diaphysis with metastasis, there is a statistically significant (*p* < .01): the number of positive cells was 8.0 ± 5.9 for 5a‐D‐Luc‐ZsGreen and 66.2 ± 26.3 for 5a‐D‐Luc‐ZsGreen‐Wnt1 (Table 3).

**TABLE 3:** Number of SATB2‐positive cells in bones.

	Normal	5a‐D‐Luc‐ZsGreen	5a‐D‐Luc‐ZsGreen‐Wnt1
Metaphysis	55.3 ± 10.0 (6)	55.3 ± 17.2 (6)	49.1 ± 26.1 (6)
Diaphysis without metastasis	11.2 ± 7.0 (6)	11.3 ± 5.9 (6)	6.5 ± 4.1 (6)
Diaphysis with metastasis^a^	‐	8.0 ± 5.9 (8)	66.2 ± 26.3 (13)**

*Note*: Data are expressed as the mean ± SD. The number of fields of view is shown in parentheses.

^a^ Metastasis means osteolytic lesions in 5a‐D‐Luc‐ZsGreen and osteoblastic lesions in 5a‐D‐Luc‐ZsGreen‐Wnt1.

** *p* < .01 (vs. 5a‐D‐Luc‐ZsGreen).

## 4 DISCUSSION

The micro‐CT images and histologic analysis of murine bones having metastasis demonstrate that overexpressed Wnt1 induces osteoblastic bone metastasis. Although the possibility that the Wnt family is associated with osteoblastic bone metastasis has been considerable,^11^ there is no direct evidence. Our current study demonstrated that Wnt1 would play a key role in osteoblastic formation. This mouse model would be a powerful tool to elucidate the molecular mechanism of osteoblastic bone metastasis.

The current study employed the breast cancer cell line 5a‐D‐Luc‐ZsGreen, forming only osteolytic bone metastasis^5^ to evaluate whether the Wnt family is involved in osteoblastic bone metastasis. Expression vectors of Wnt1, Wnt3A, and Wnt5A were introduced into the cell line, and eight stably expressing subclones for each gene were established. The highest expression cell clone for each gene was inoculated into the left cardiac ventricle. CT imaging of metastasized bones found an osteoblastic change in bones having Wnt1‐expressing tumors (5a‐D‐Luc‐ZsGreen‐Wnt1) but not in bones having Wnt3A‐ or Wnt5A‐expressing tumors. That was confirmed by H&E staining: osteoblastic change was observed only in Wnt‐1‐expression tumors, where differentiated osteoblasts were observed by SATB2 immunohistostaining analysis. These findings indicate that Wnt1 is involved in osteoblastic bone metastasis.

SATB2 is a differentiation marker for osteoblasts,^24^, ^25^ the upregulation actives Wnt/β‐catenin signaling, and the knockdown decreased the activity.^26^ SATB2‐positive cells at 5a‐D‐Luc‐ZsGreen‐Wnt1 osteoblastic bone metastasis sites were observed approximately eight times higher than those at 5a‐D‐Luc‐ZsGreen osteolytic lesions with a statistical significance of *p* < .01. Taken together, it suggests that overexpression of Wnt1 activated SATB2, which in turn activated the downstream Wnt/β‐catenin signaling and promoted osteogenesis.

The quantitative analysis of SATB2 also showed that in bones with 5a‐D‐Luc‐ZsGreen‐Wnt1 osteoblastic lesions, the number of positive cells in the metaphysis and non‐metastasized diaphysis was slightly decreased compared to normal and 5a‐D‐Luc‐ZsGreen mice: by more than 10% in the metaphysis and by more than 40% in the diaphysis without metastasis. There is an interesting finding in a clinical SPECT/CT study in prostate cancer patients: ^99m^Tc‐methylene diphosphonate (MDP) uptake was significantly lower in non‐metastatic sites in prostate cancer patients with bone metastases than in patients without metastases.^27^ ^99m^Tc‐MDP uptake is associated with osteoblast maturation and mineralization.^28^ The lower uptake observed in the clinical study could reflect fewer mature osteoblasts as in our preclinical model.

Wnt3A belongs to the β‐catenin pathway like Wnt1.^11^ It has been reported that overexpression of Wnt3A in bone has a marked inhibitory effect on myeloma‐induced osteolytic bone lesions.^13^ The current study observed a similar extent of osteolytic images in Wnt3A‐overexpressing cell transplantation to in the parental 5a‐D‐Luc‐ZsGreen tumors. The reason why Wnt3A induced no osteoblastic change is unclear. Further studies are required to address whether Wnt3A is also involved in osteoblastic change in other cancer than myeloma.

Wnt5A is on the β‐catenin‐independent pathway.^11^ There are reports that Wnt5A expressed from osteoblasts binds to the FZD‐ROR2 receptor complex and stimulates osteoclast precursor differentiation.^29^ The role of Wnt5A in bone metabolism is different from Wnt1 and Wnt3A, suggesting that this pathway is not associated with an osteoblastic change in bone metastasis.

Our findings are in line with clinical findings about bone metabolic markers.^30^, ^31^ In the blood of prostate cancer patients with bone metastasis, there is an increase in bone‐type alkaline phosphatase, a marker of bone formation, but a limited enhancement of osteocalcin.^30^, ^31^ Rawadi et al. reported that the Wnt signaling pathway activation induced alkaline phosphatase gene expression, but not induced the osteocalcin gene.^32^ These findings suggest Wnt1 is involved in osteoblastic bone metastasis in prostate cancer patients although further clinical studies are required.

It has been reported that Wnt hyperactivity is limited in prostate cancer, but the expression of the Wnt antagonist sclerostin is also low.^33^ Sclerostin inhibition increases bone mass.^34^ These findings suggest that the Wnt signaling activation through sclerostin inhibition is associated with osteogenic bone metastasis development in prostate cancer. The fact that many drugs targeting the Wnt signaling pathway have been developed and several are already approved could promptly lead to clinical studies. That encourages further clinical studies with anti‐Wnt drugs to confirm our findings in the present study.

There are three limitations in the present study. First, parental 5a‐D‐Luc‐ZsGreen cells induce extensive bone loss. This could lead to an underestimation of the number of osteoblasts in the osteolytic metastatic sites. It is necessary for further studies with a new bone metastatic model not showing osteolytic change. Second, the effects of Wnt1 inhibition on osteoblastic changes were not evaluated in 5a‐D‐Luc‐ZsGreen‐Wnt1 mice. Wnt inhibitors generally affect tumor cell survival. It is difficult to distinguish between the effects of Wnt inhibition and cytoreduction. There is a need to develop a method that only inhibits the Wnt pathway but does not decrease cells. Finally, only the downstream molecule SATB2 was evaluated. SATB2 directly interacts with the activity of Runx2 and ATF4, which regulate osteoblast differentiation.^25^ Further studies evaluating additional downstream molecules may provide deeper insight into the mechanism of osteoblastic skeletal metastasis.

In summary, our data showed that overexpression of Wnt1 induced osteoblastic change in an osteolytic breast cancer model. It suggests that Wnt1 is an important factor in osteogenesis. Our osteoblastic bone metastasis model could be a powerful tool to promote bone metastasis treatment research.

## AUTHOR CONTRIBUTIONS

**Aya Sugyo:** Data curation (equal); funding acquisition (equal); investigation (equal); methodology (equal); writing – original draft (equal). **Atsushi B. Tsuji:** Conceptualization (equal); data curation (equal); funding acquisition (equal); investigation (equal); writing – original draft (equal); writing – review and editing (lead). **Hitomi Sudo:** Funding acquisition (equal); investigation (equal); methodology (equal); validation (equal). **Yoshiya Sugiura:** Data curation (equal); methodology (equal). **Mitsuru Koizumi:** Conceptualization (lead); supervision (equal); writing – review and editing (equal). **Tatsuya Higashi:** Conceptualization (equal); supervision (equal); writing – review and editing (equal).

## CONFLICT OF INTEREST STATEMENT

The authors have stated explicitly that there are no conflicts of interest in connection with this article.

## ETHICS STATEMENT

The animal experiments were approved by the Institutional Animal Care and Use Committee of the National Institutes for Quantum Science and Technology, and all animal experiments were conducted by the institutional guidelines regarding animal care and handling.

## Supporting information


**Supplementary Figure S1.** Micro‐CT images of 13 mice showing osteoblastic lesions after 5a‐D‐Luc‐ZsGreen‐Wnt1 cell inoculation at the 2nd experiment. Yellow arrowheads indicate osteoblastic lesions.

**Supplementary Figure S2.** SATB2 immunostaining of a normal mouse bone.

## Data Availability

The data that support the findings of this study are available from the corresponding author upon reasonable request.
