# Supplementary figures and images for: Wnt1 induces osteoblastic changes in a well‐established osteolytic skeletal metastatic model derived from breast cancer
Source: Cancer Rep (Hoboken). 2023 Oct 15;6(12):e1909. doi: 10.1002/cnr2.1909 (PMC10728502; doi:10.1002/cnr2.1909)

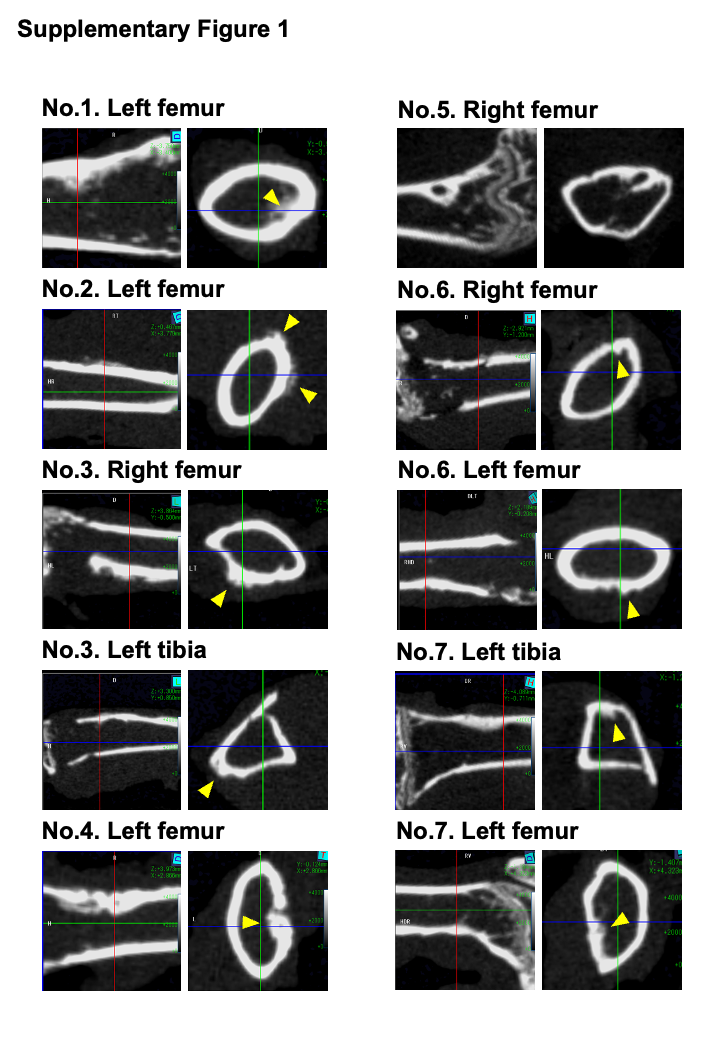

Supplement: Supplementary file 1 — Supplementary Figure S1. Micro‐CT images of 13 mice showing osteoblastic lesions after 5a‐D‐Luc‐ZsGreen‐Wnt1 cell inoculation at the 2nd experiment. Yellow arrowheads indicate osteoblastic lesions. [file CNR2-6-e1909-s002.zip › SupplFig1.tiff]

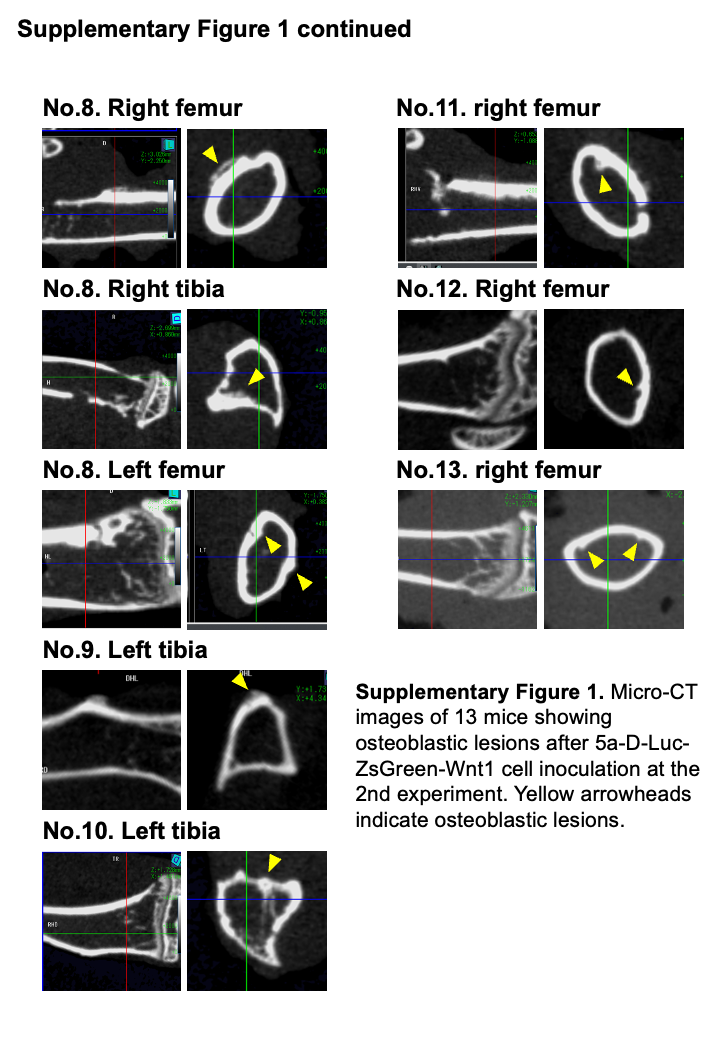

Supplement: Supplementary file 1 — Supplementary Figure S1. Micro‐CT images of 13 mice showing osteoblastic lesions after 5a‐D‐Luc‐ZsGreen‐Wnt1 cell inoculation at the 2nd experiment. Yellow arrowheads indicate osteoblastic lesions. [file CNR2-6-e1909-s002.zip › SupplFig1cont.tiff]

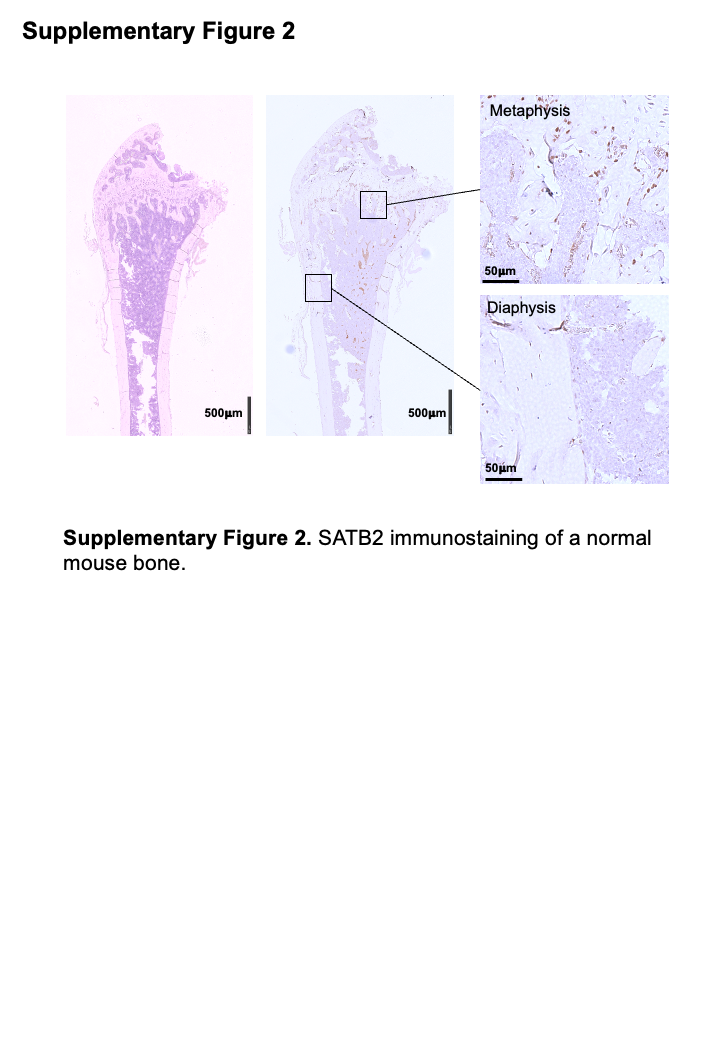

Supplement: Supplementary file 2 — Supplementary Figure S2. SATB2 immunostaining of a normal mouse bone. [file CNR2-6-e1909-s001.tiff]
